# MicroRNA-212 Post-Transcriptionally Regulates Oocyte-Specific Basic-Helix-Loop-Helix Transcription Factor, Factor in the Germline Alpha (FIGLA), during Bovine Early Embryogenesis

**DOI:** 10.1371/journal.pone.0076114

**Published:** 2013-09-27

**Authors:** Swamy K. Tripurani, Gabbine Wee, Kyung-Bon Lee, George W. Smith, Lei Wang, JianboYao

**Affiliations:** 1 Laboratory of Animal Biotechnology and Genomics, Division of Animal and Nutritional Sciences, West Virginia University, Morgantown, West Virginia, United States of America; 2 Laboratory of Mammalian Reproductive Biology and Genomics, Michigan State University, East Lansing, Michigan, United States of America; 3 Department of Animal Science, Michigan State University, East Lansing, Michigan, United States of America; 4 Department of Physiology, Michigan State University, East Lansing, Michigan, United States of America; McGill University, Canada

## Abstract

Factor in the germline alpha (FIGLA) is an oocyte-specific basic helix-loop-helix transcription factor essential for primordial follicle formation and expression of many genes required for folliculogenesis, fertilization and early embryonic survival. Here we report the characterization of bovine *FIGLA* gene and its regulation during early embryogenesis. Bovine *FIGLA* mRNA expression is restricted to gonads and is detected in fetal ovaries harvested as early as 90 days of gestation. *FIGLA* mRNA and protein are abundant in germinal vesicle and metaphase II stage oocytes, as well as in embryos from pronuclear to eight-cell stage but barely detectable at morula and blastocyst stages, suggesting that *FIGLA* might be a maternal effect gene. Recent studies in zebrafish and mice have highlighted the importance of non-coding small RNAs (microRNAs) as key regulatory molecules targeting maternal mRNAs for degradation during embryonic development. We hypothesized that *FIGLA*, as a maternal transcript, is regulated by microRNAs during early embryogenesis. Computational predictions identified a potential microRNA recognition element (MRE) for miR-212 in the 3’ UTR of the bovine *FIGL*A mRNA. Bovine miR-212 is expressed in oocytes and tends to increase in four-cell and eight-cell stage embryos followed by a decline at morula and blastocyst stages. Transient transfection and reporter assays revealed that miR-212 represses the expression of FIGLA in a MRE dependent manner. In addition, ectopic expression of miR-212 mimic in bovine early embryos dramatically reduced the expression of FIGLA protein. Collectively, our results demonstrate that FIGLA is temporally regulated during bovine early embryogenesis and miR-212 is an important negative regulator of FIGLA during the maternal to zygotic transition in bovine embryos.

## Introduction

During oocyte growth and follicular development, oocytes accumulate maternal effect factors necessary for oocyte maturation, fertilization and early embryogenesis [1]. Identification and characterization of maternal (oocyte-derived) genes would be extremely useful in unraveling their specific functions in fertilization, early embryogenesis and preimplantation development [2]. Recently, studies from our research group and several others demonstrated that genes that are specifically expressed in oocytes and play an important role in oocyte growth during follicular development are also essential during early embryonic development and critical regulators of embryonic genome activation, pluripotency gene expression and blastocyst cell allocation [3]. However, the identities and functions of many more key oocyte-specific genes involved in the above process are relatively unknown, especially in farm animal species.

FIGLA is an oocyte-specific basic helix-loop-helix transcription factor, and it was originally identified through its involvement in the coordinate expression of genes encoding glycoproteins (*Zp1, Zp2 and Zp3*) that form the extracellular matrix that surrounds growing oocytes, ovulated eggs and pre-implantation embryos [4]. FIGLA heterodimerizes with a β-helix-loop-helix E12 protein and binds to canonical E-box motifs in the promoter region of all three mouse zona pellucida genes [4]. To date, functional homologues of FIGLA have been identified in mice, human and zebrafish [4–8]. *Figla* transcript is expressed in mouse embryonic gonad as early as E15.5, and then undergoes a dramatic increase at the end of embryonic development to peak by postnatal day 2 - a time when the oocyte has become enclosed in primordial follicles and persists throughout folliculogenesis [5]. A similar temporal expression was observed in humans, with *FIGLA* transcript increasing between 17-19 weeks of gestation (precise time at which primordial follicles are first formed) [9]. Female mice lacking FIGLA are infertile whereas males have normal fertility. Although germ cell migration and proliferation during embryonic gonadogenesis appeared normal, primordial follicles were not formed at birth, and massive depletion of oocytes occurred within a few days after birth resulting in shrunken ovaries and female sterility in *Figla* null mice [5]. Recently, two plausible mutations in the *FIGLA* gene have been found in patients with premature ovarian failure (a heterogeneous genetic disorder characterized by premature depletion of ovarian follicles before the age of 40) [10]. Further, comparison of gene expression patterns between wild type and *Figla* knockout mice ovaries by microarray and serial analysis of gene expression (SAGE) have identified several oocyte-specific genes, including maternal effect genes, that are directly or indirectly regulated by FIGLA [11]. In addition, recently it was shown that physiological expression of FIGLA also plays a critical dual role in activation of oocyte-associated genes and repression of sperm-associated genes during normal postnatal oogenesis [12]. These observations indicate that FIGLA plays a key regulatory role in female germline follicle development, fertilization and early embryogenesis. However, despite its established role in control of oocyte gene expression, the temporal expression and regulation of FIGLA during early embryogenesis have not been investigated.

As embryonic development proceeds and becomes dependent on expression of embryonic genes, maternally inherited RNAs and proteins are depleted [1,13]. Degradation of maternally derived mRNAs and proteins in coordination with embryonic genome activation (EGA) is experimentally proven to be a crucial checkpoint during early embryo development in mouse [14,15] and Xenopus [16]. Although the mechanisms responsible for this RNA degradation are incompletely understood, recent studies in zebrafish [17,18], Xenopus [19] and mouse [20,21] have implicated microRNAs in regulating this evolutionary conserved mechanism of vertebrate development. MicroRNAs (miRNAs) comprise a large family of small single stranded non-coding RNAs that are evolutionarily conserved, endogenous, and 21–23 nucleotides in length [22,23]. It is predicted that miRNAs account for 1-5% of the human genome and target ~60% of human mRNAs [22]. miRNAs normally function as negative gene regulators at the post-transcriptional level by binding to the 3′UTRs of the target mRNA through base pairing, resulting in its cleavage or translation inhibition [23]. Although little is currently known about the specific targets and biological functions of miRNA molecules thus far, increasing evidence indicates that miRNAs play crucial roles in the regulation of gene expression controlling a variety of basic cellular, biological and pathological processes, such as metabolism, proliferation, development, differentiation, apoptosis and oncogenesis [24]. Since early embryo development consists of a highly choreographed series of events controlled by temporally and spatially regulated batteries of genes, it is thus conceivable that miRNAs also play a pivotal role in early embryogenesis.

In recent years, farm animals such as cattle, sheep and pig have been recognized as important animal models for biomedical research [25]. Especially, due to the similarities in folliculogenesis, ovulation and number of embryonic cell cycles required for embryonic genome activation during early embryogenesis between humans and cattle compared to the traditional animal model (mouse), the bovine model provides an indispensable research platform for understanding the reproductive physiology and diseases and disorders associated with it, such as infertility, ovarian cancer and early pregnancy loss [26,27]. Furthermore, several assisted reproductive techniques such as superovulation, oocyte culturing, in-vitro fertilization and embryo maturation, transfer and freezing that are most commonly used in humans were developed based upon many years of research with bovine embryos [27]. For these reasons, comparative genomic approaches coupled to functional studies in non-traditional models are necessary to provide information on existence of genes that play essential role in fertility in non-murine models such as humans. Here, we report the cloning of the bovine orthologue of *FIGLA* gene, the characterization of its mRNA and protein expression during bovine oocyte maturation and early embryonic development, and the demonstration of miRNA-212 as a potential negative regulator of bovine FIGLA during bovine early embryogenesis.

## Materials and Methods

### Tissue collection

Bovine tissue samples, including adult liver, lung, thymus, kidney, muscle, heart, spleen, cortex (brain), pituitary, adrenal, testis, ovary, and fetal testis and ovaries, were collected at a local slaughterhouse (JBS Packerland, Plainwell, MI) with permission obtained from the slaughterhouse for the use of these tissues in research. All samples were frozen in liquid nitrogen and stored at -80 °C until use.

### RNA preparation, cDNA synthesis and RT-PCR analysis

Total RNA from different bovine tissues and fetal ovary samples collected from a local slaughterhouse as described previously [28] were extracted using TRIzol reagent (Invitrogen) according to the manufacturer’s instructions. Two micrograms of DNase-treated total RNA from various bovine tissue and fetal ovary samples were reverse transcribed to first-strand cDNA using Superscript III reverse transcriptase (Invitrogen) according to the manufacturer’s instructions. The RT-PCR was performed by denaturation at 95 °C for 3 min followed by 35 cycles of 95 °C for 30 sec, 58 °C for 45 sec, and 72 °C for 90 sec and final extension at 72 °C for 10 min. Bovine ribosomal protein L19 (*RPL19*) was used as a positive control for RNA quality and RT (See Table S1 for primers used for RT-PCR analysis).

### cDNA cloning and Northern blot analysis of bovine FIGLA

Cloning of bovine *FIGLA* was performed as described in [3,28,29] with minor modifications. Briefly, based on the predicted sequence for the bovine *FIGLA* gene available in the National Centre for Biotechnology Information (NCBI) database, primers were designed (Table S1) to amplify a fragment in the predicted ORF of the bovine *FIGLA* gene from a bovine fetal ovary sample. The amplified product (468 bp) was cloned using the TOPO® TA cloning kit (Invitrogen) and sequenced. Based on the obtained bovine *FIGLA* cDNA sequence, primers for 5’ and 3’ RACE were designed (Table S1). RACE experiments were performed to extend the 5’ and 3’ ends of bovine *FIGLA* cDNA using the second generation 5’/3’ RACE kit (Roche Diagnostics) following the manufacturer’s protocol. To determine the size of bovine *FIGLA* transcript, a Northern blot was performed as described previously [28] with mRNA isolated from an adult ovary sample.

### Quantification of bovine FIGLA mRNA and protein in oocytes and early embryos

Quantitative measurement of *FIGLA* mRNA expression in oocytes and early embryos was performed as described previously [3,28,29]. Briefly, the oocytes and embryo samples used in the experiment included GV and MII stage oocytes and pronuclear, 2-cell, 4-cell, 8-cell, 16-cell, morula and blastocyst stage embryos (n=5 pools of 10 embryos) generated by *in vitro* fertilization of abattoir-derived oocytes as described elsewhere [30]. Total RNA from oocytes and embryos was isolated using the RNA-queous®-Micro kit (Ambion). Before RNA extraction, each sample was spiked with 250 fg of green fluorescent protein (GFP) synthetic RNA (polyadenylated) as an exogenous control. The quantity for *FIGLA* mRNA was normalized relative to the quantity of *GFP* RNA measured in each sample to account for variations in RNA recovery and efficiency of cDNA synthesis between samples (See Table S1 for primers used in the analysis).

To determine the expression of bovine FIGLA protein in oocytes and early embryos, western blot analysis was performed as previously described [28]. The primary antibody (anti-bovine FIGLA) was prepared commercially (GenScript Corporation) by immunizing rabbits with a 15 amino acid synthetic peptide (CKRDPDHQSYSSNTS) of bovine FIGLA protein. As a loading control, β-Actin mouse monoclonal antibody (Santa Cruz; SC- 47778) was used.

### miRNA-212 binding site prediction and expression analysis

Potential miRNA binding sites in the 3’ UTR of bovine *FIGLA* mRNA were predicted using MicroInspector, an algorithm for detection of possible interactions between miRNAs and target mRNA sequences [31]. The expression of miRNA-212 in multiple tissues, oocytes and early embryos was performed as described previously [32] (See Table S1 for primers used in the analysis).

### Plasmids, cell transfection and luciferase reporter assays

The plasmid expressing bovine FIGLA (pcDNA3.1: FLAG-FIGLA) was constructed by cloning the bovine *FIGLA* cDNA (open reading frame plus 3’ UTR of isoform 1) in frame with a FLAG tag sequence at 5` end into pcDNA3.1 expression vector (Invitrogen). miR-212 expression plasmid (pcDNA3.1: miR-212) was constructed by cloning a 220 bp fragment (surrounding pre-miR-212) amplified from bovine genomic DNA. Both constructs were sequenced to ensure that no mutations were introduced during PCR amplification. For construction of luciferase reporter plasmids, *FIGLA* 3′UTR was cloned into pmirGLO Dual-Luciferase miRNA target expression vector (Promega). Mutation of the miR-212 miRNA recognition element (MRE) in the *FIGLA* 3’ UTR was performed using the QuikChange site-directed mutagenesis kit (Stratagene) according to the manufacturer’s instructions. Primers used for plasmid constructions are listed in Table S1.

HeLa cells were maintained in DMEM media supplemented with 10% FBS, 100 µg/ml streptomycin, and 100 U/ml penicillin (Gibco), and cultured at 37°C and 5% CO_2_. For transient transfection, FuGENE6 (Roche Applied Science) was used according to the manufacturer’s instructions. Following transfection, cells were incubated for 48 h before harvest for western blot analysis and luciferase assay. Western blot analysis of FIGLA protein expression in the transfected cells was performed using anti-FLAG antibody (Sigma). Detection of β-Actin (protein loading control) was performed using anti-β-Actin monoclonal antibody (Santa Cruz; SC- 47778). Luciferase assay was performed using the Dual-Glo luciferase assay system (Promega) following the instructions as described by the manufacturer. Firefly luciferase activity was normalized to renilla luciferase activity to adjust for variations in transfection efficiency among experiments. All transfection experiments were performed in quadruplicate (n = 4) with data averaged from four independent experiments.

### Microinjection experiments

*In vitro* maturation of oocytes (obtained from abattoir-derived ovaries), *in vitro* fertilization to generate zygotes, microinjection of miRNA mimic into zygotes and subsequent embryo culture were conducted using procedures described previously [30,33]. Briefly, each *in vitro* fertilized one-cell embryo was microinjected with ~20 picoliters of miRNA mimic using an inverted Nikon microscope equipped with micromanipulators (Narishige International USA, Inc., East Meadow, NY). Mature miRNA-212 mimic (MIMAT0022695) and a negative control miRNA mimic (cel-miR-67, CN- 001000-01-05) were obtained from Dharmacon Technologies (Dharmacon Inc, Lafayette, CO), and diluted with RNase free water to a final concentration of 20 µM before microinjection. Uninjected embryos and embryos injected with the negative control miRNA mimic were used as control groups. Each group contained 20 embryos per replicate (n = 2). The efficiency of FIGLA protein knockdown was determined by immunocytochemistry in eight-cell stage embryos as described previously. Imaging was performed using confocal spinning-disk microscopy. Optical sections (every 1 µm) were acquired for each embryo and MetaMorph software (Universal Imaging, Downingtown, PA, USA) was used for image acquisition and analysis.

## Results and Discussion

### cDNA cloning, genome organization and expression of bovine FIGLA

**Figure 1:**
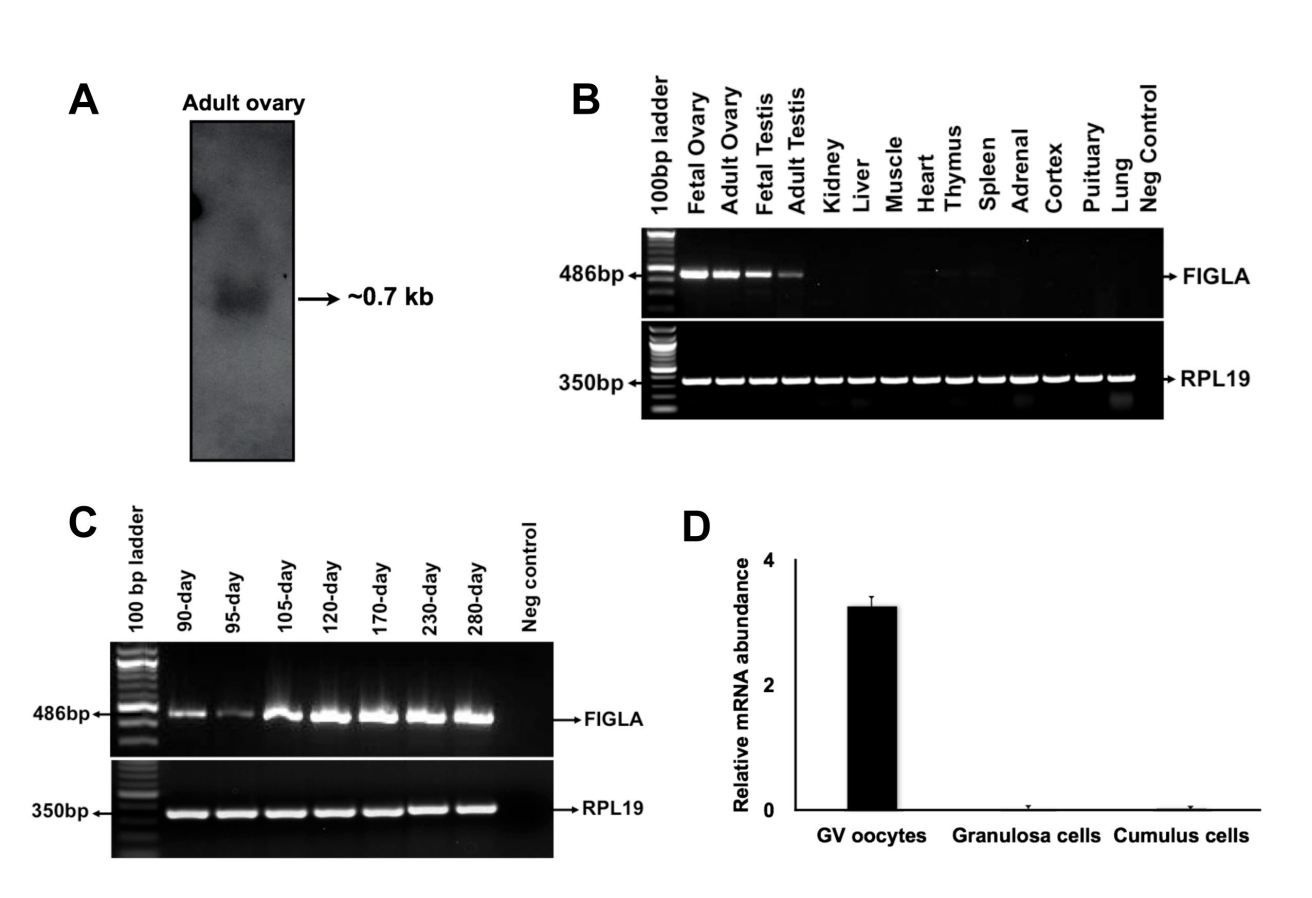
Analysis of bovine *FIGLA* mRNA expression. (A) Northern blot analysis of *FIGLA* transcript. (B) Expression of *FIGLA* mRNA in various bovine tissues. (C) Expression of *FIGLA* mRNA in bovine fetal ovaries of different developmental stages. (D) Quantitative expression of *FIGLA* mRNAs in granulosa cells, cumulus cells and GV oocytes. Data were normalized relative to abundance of endogenous control ribosomal protein S18 (RPS18) and are shown as mean + SEM (n=3).

A cDNA fragment (468 bp) representing part of the predicted coding region of bovine *FIGLA* mRNA was successfully amplified from a bovine fetal ovary cDNA sample using the primers designed based on the predicted bovine *FIGLA* cDNA sequence (XM_607327.3). Northern blot analysis detected a single transcript of approximately 0.7 kb in bovine adult ovary samples (Figure 1A). Using RACE and RT-PCR, two different cDNA sequences corresponding to two isoforms of bovine *FIGLA* mRNA were obtained (Figure S1A). The longer isoform (isoform 1, 660 bp, KF026766) contains an open reading frame encoding a protein of 165 amino acids. The shorter isoform (isoform 2, 630 bp, KF026767), which results from alternative splicing of exon 4, encodes a protein of 171 amino acids. The two isoforms were not distinguishable by Northern blot analysis. However, RT-PCR analysis using primers flanking exon 4 confirmed the presence of two isoforms in oocytes and fetal ovaries (Figure S1B). Both isoforms contain a conserved basic helix-loop-helix domain (Figure S2). A blast search of the bovine genome database at NCBI revealed that the bovine *FIGLA* gene is located on chromosome 11 and spans approximately 14.7 kb. The bovine *FIGLA* gene has 5 exons and 4 introns (Table S2), and all splice sites are in agreement with the consensus sequence (GT-AG rule).

Analysis of tissue distribution by semi-quantitative RT-PCR revealed that bovine *FIGLA* expression is restricted to fetal and adult ovaries with minor expression in adult testis samples (Figure 1B). Expression analysis in fetal ovaries of different developmental stages revealed that bovine *FIGLA* is expressed as early as 90 days of gestation period (when primordial follicles start to form in cows) and is highly abundant in the fetal ovaries of late gestation (Figure 1C). A similar timing of *FIGLA* expression during primordial follicle formation is also found in humans [9] and mouse [5], indicating an evolutionarily conserved role during mammalian folliculogenesis. Further, quantitative real-time PCR analysis using RNA isolated from oocytes, granulosa cells, and cumulus cells indicates that bovine *FIGLA* is exclusively expressed in oocytes but not in the surrounding follicular somatic cells (Figure 1D).

### Expression of bovine FIGLA mRNA and protein during oocyte maturation and early embryonic development

**Figure 2:**
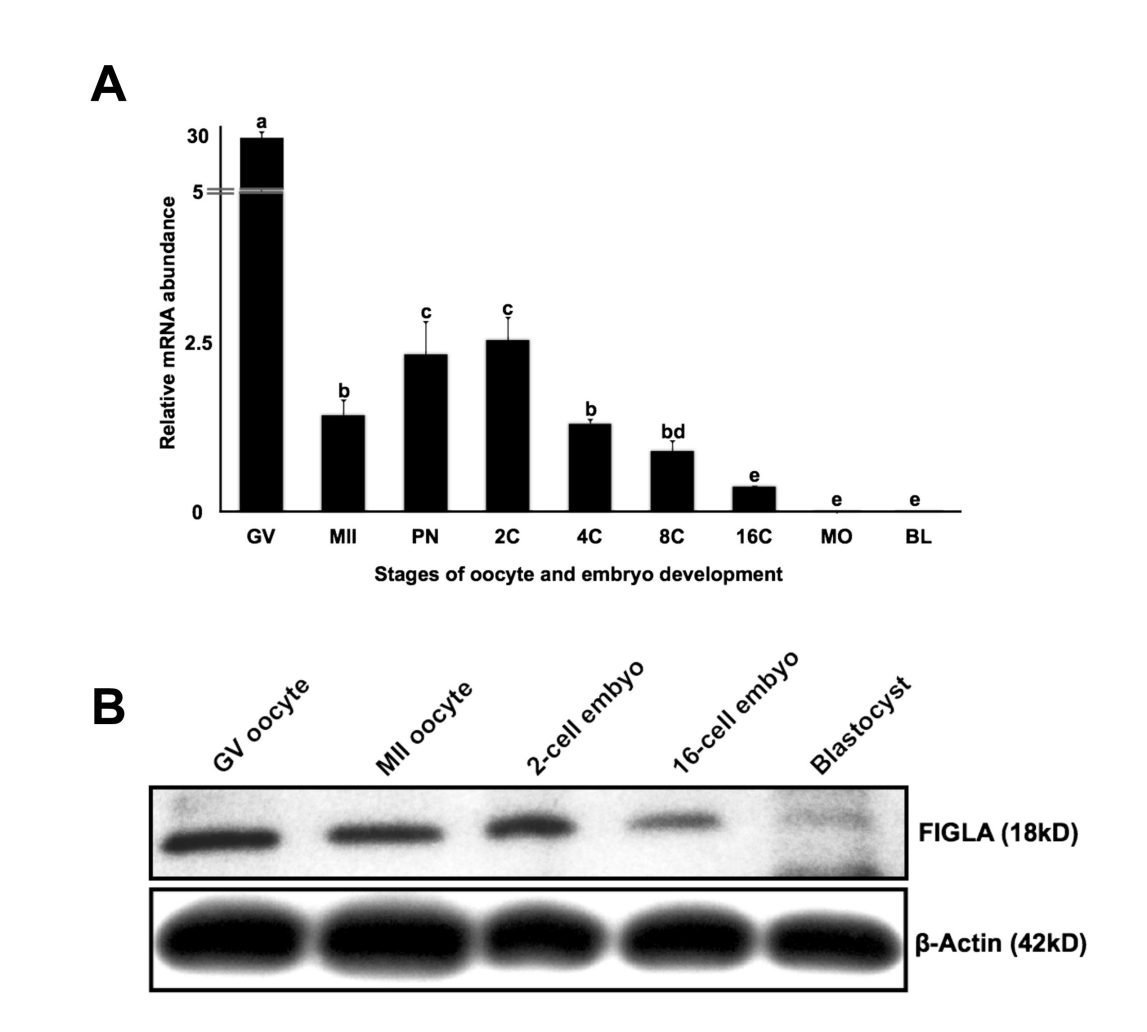
Expression of *FIGLA* mRNA and protein during oocyte maturation and early embryonic development. (A) Analysis of steady-state levels of *FIGLA* mRNA in *in* *vitro* produced bovine pre-implantation stage embryos. The oocyte and embryo samples used in this experiment included GV and MII stage oocytes, pronuclear (PN), 2-cell (2C), 4-cell (4C), 8-cell (8C), 16-cell (16C), morula (MO) and blastocyst (BL) stage embryos. Data were normalized relative to abundance of exogenous control (GFP) RNA and are shown as mean + SEM (n = 4 pools of 10 embryos per stage). Different letters indicate statistical difference (P < 0.05). (B) Western blot analysis of FIGLA protein expression in bovine oocytes and early embryos using antibodies specifically against bovine FIGLA (25 oocytes or embryos per lane).

To demonstrate the temporal expression of bovine FIGLA during preimplantation embryo development, real-time PCR analysis was performed. The expression pattern of bovine *FIGLA* mRNA during early embryogenesis is similar to several known maternal effect genes such as *NOBOX* [3], *MATER* [34] and *ZAR1* [35] (Figure 2A), showing dynamically regulated expression during the window from meiotic maturation through embryonic genome activation. Western blot analysis demonstrated that the size of the FIGLA protein is ~ 18 kD and that the protein is abundant in GV and MII stage oocytes as well as in 2-cell stage embryos, but the expression drops by the 16-cell stage embryos and is barely detectable at the blastocyst stage (Figure 2B). Overall, the expression pattern of bovine FIGLA, suggests a potential role as a maternal effect factor during early embryonic development.

The maternal-to-embryonic transition (MET) is a key event in early embryonic development, which occurs at 2-cell stage in mouse, and much later in domestic ruminants (8-16 cell stage in cattle and sheep) [36]. Gene-targeting studies in mice have demonstrated that maternal-effect genes such as *Mater*, *Zar1* and *Npm2* are transcribed and stored during oogenesis and play an essential role during early embryogenesis by mediating MET. Such direct experimental evidence is lacking to support the requirement of the above genes during bovine early embryogenesis. Recently, we provided the first evidence of a novel and unrecognized functional role for the oocyte-specific transcription factor newborn ovary homeobox (NOBOX) during bovine early embryogenesis. In addition to its role in regulating many germ-cell specific transcripts during folliculogenesis in mice, NOBOX is temporally regulated during early embryonic development and functions in regulating several genes involved in cell cycle progression, transcriptional regulation, signal transduction and epigenetic modification during embryonic genome activation (EGA), pluripotency gene expression and blastocyst cell allocation [3]. In addition, morpholino-mediated depletion of germ-cell specific transcription factor essential for primordial germ cell survival, *Pou5f1/Oct4* in mouse one-cell embryos affected embryonic development and expression of transcriptional and post-transcriptional regulators during MET [37]. These studies indicate that transcripts expressed during folliculogenesis also play a pivotal role during oocyte maturation and embryonic development. Furthermore, serial analysis of gene expression in *Figla* null newborn ovaries revealed misregulation of several genes required for folliculogenesis, including maternal genes such as *Pou5f1/Oct4* [37], *Dppa3* [38] and *Padi6* [39] essential for embryonic development. Taken together, FIGLA, a germ-cell specific maternal effect transcription factor, might play a pivotal role in modulating multiple genetic hierarchies involved in folliculogenesis and pre-implantation development.

### Prediction of miRNA binding site in the 3`UTR of bovine FIGLA mRNA

**Figure 3:**
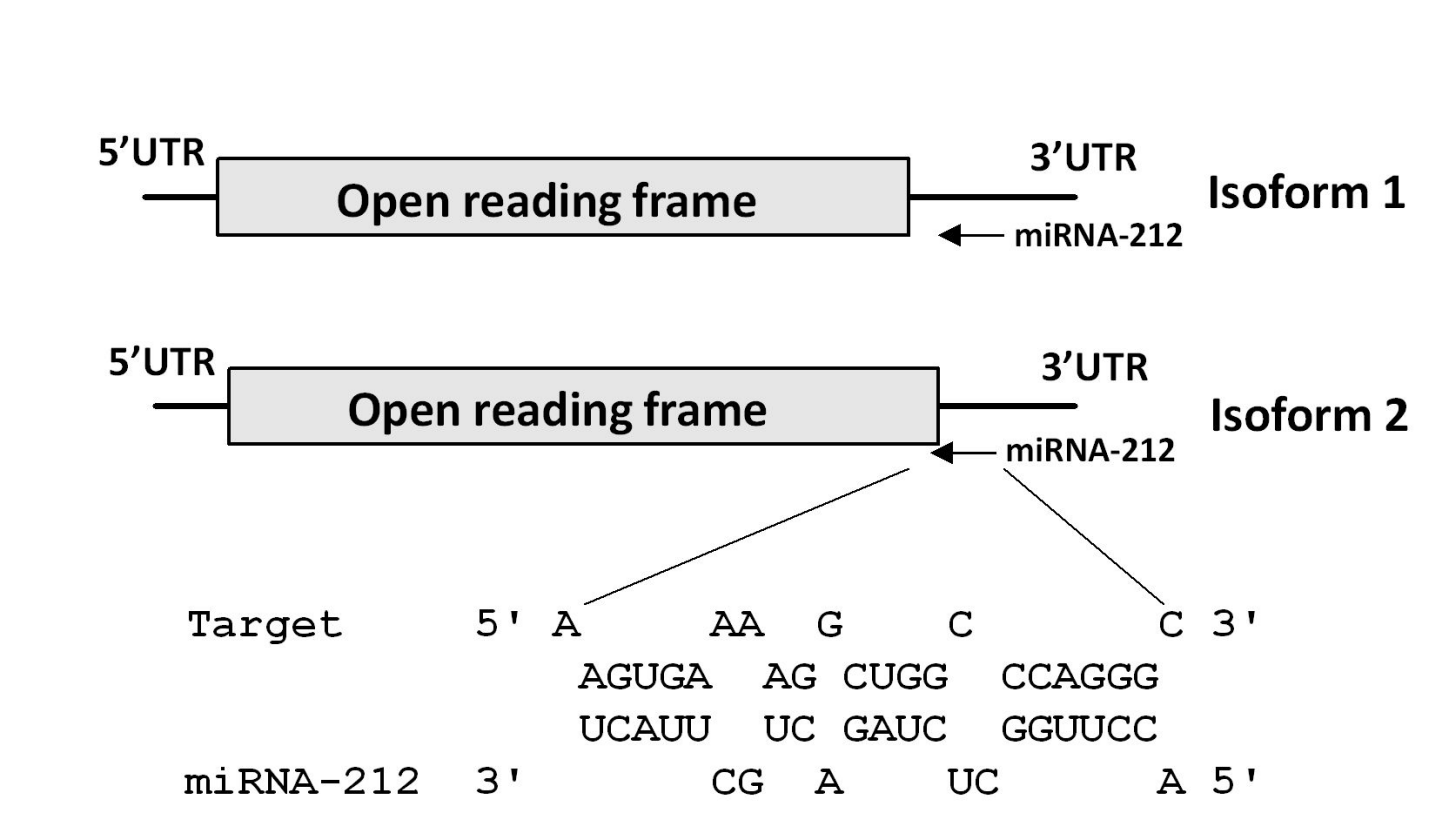
Prediction of a miR-212 binding site in the 3`UTR of bovine *FIGLA* mRNA. The prediction was performed using MicroInspector. Both *FIGLA* isoforms contain the binding site.

During the last two decades, miRNAs emerged as critical regulators of many cellular, developmental and physiological processes, including the pathogenesis of several diseases [24]. Recently, miRNAs have been demonstrated to degrade/regulate key maternal RNA transcripts and proteins during early embryonic development in zebrafish [18], Xenopus [19] and mouse [21]. To examine the possibility of FIGLA regulation by miRNAs, we searched for potential miRNA-binding sites in the 3`UTR region of *FIGLA* mRNA using the “Microinspector” algorithm [31]. The computational search identified a miRNA recognition element (MRE) for miR-212 in bovine *FIGLA* 3`UTR. The identified MRE has a low predicted free energy of hybridization with miR-212 (−20.86 kcal/mol), suggesting a stable miR:MRE duplex within the 6 nt seed region at the 5` end of the miRNA (Figure 3). Several reports have demonstrated that the seed region is the most important region of miRNA:mRNA interaction and a key determinant of miRNA-induced repression of gene expression [40]. Furthermore, secondary structure analysis revealed that the apparent miR-212 target site was positioned on a hairpin-loop structure in an exposed position, which might facilitate miRNA accessibility. These findings suggest that miR-212 is likely an important regulator of FIGLA.

### Expression analysis of miR-212

**Figure 4:**
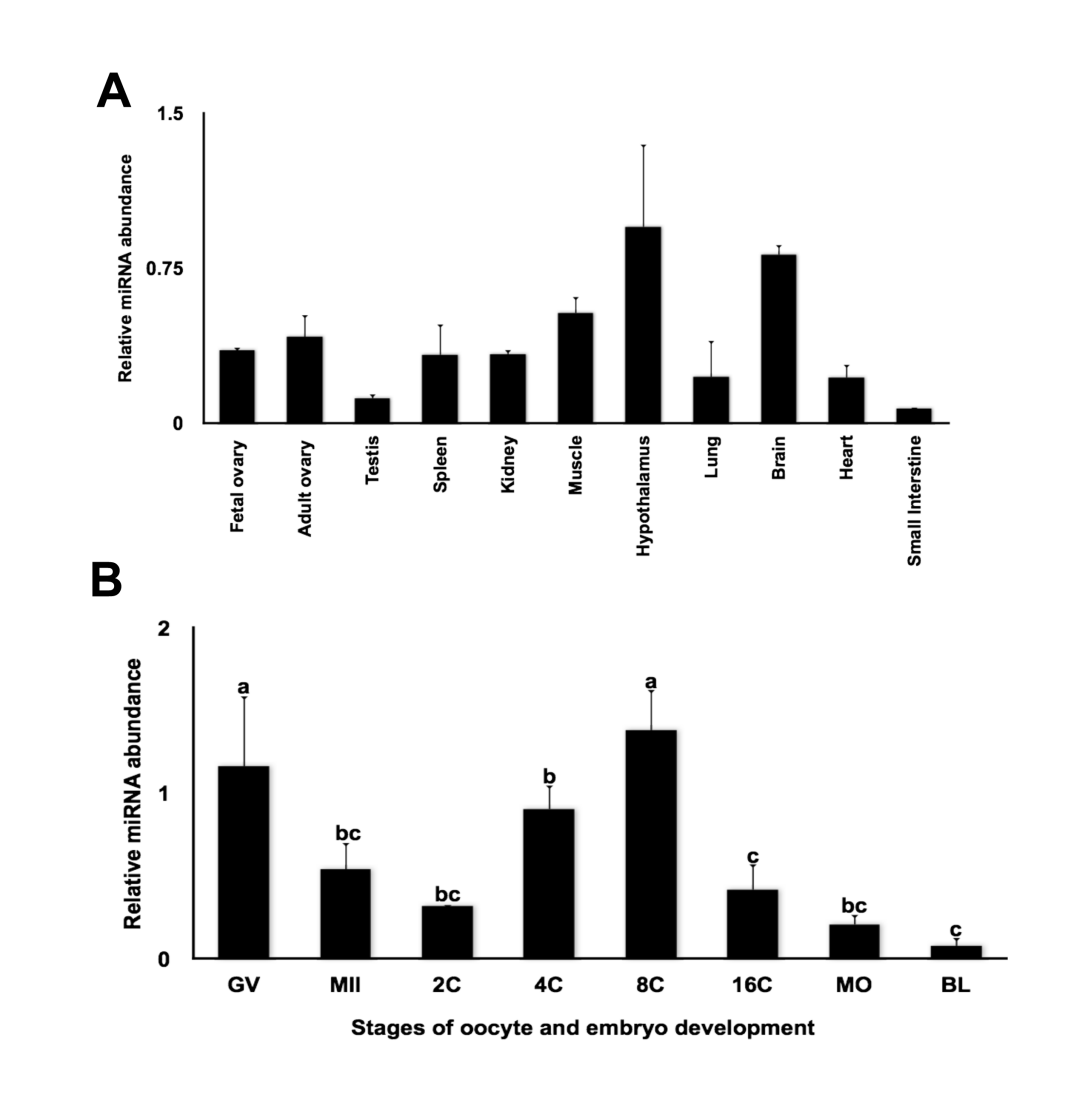
Expression analysis of miR-212. (A) Tissue distribution of miR-212 analyzed by quantitative real time PCR. Data were normalized relative to abundance of endogenous control ribosomal protein S18 (RPS18) and abundance expressed as relative fold change using the sample with the lowest value as the calibrator (mean ± SEM; n = 4 per tissue). (B) Relative abundance of miR-212 in bovine oocytes and *in* *vitro* produced bovine embryos. Data were normalized to abundance of miR-125b mRNA and expressed as relative fold change using the sample with the lowest value as the calibrator (mean ± SEM; n = 4 pools of five oocytes/embryos each). Different letters indicate statistical difference (P< 0.05).

miR-212 arises from the miR-212/132 cluster (which comprises miR-212 and miR-132). It is transcribed from a stable intron of a non-coding transcript localized on chromosome 11 in mice [41], chromosome 17 in humans [42] and chromosome 19 in cattle. miR-212 and miR-132 are closely related as they have identical seed sequences and the mature miRNA differs only by four nucleotides. To determine the expression profiles of miRNA-212 in bovine tissues and embryos, quantitative real-time PCR was performed. MiR-212 was predominantly expressed in hypothalamus and brain (Figure 4A), which is consistent with the expression profile observed in mice [43]. In addition, expression of miR-212 in fetal and adult ovary was also detected (Figure 4A). Expression analysis during oocyte maturation and early embryonic development showed that miR-212 is expressed in GV oocytes and tends to increase at the 4-cell and 8-cell stage embryos followed by a decline at morula and blastocyst stages (Figure 4B). A similar expression pattern was observed for miR-430 in zebrafish [18], miR-427 in Xenopus [19] and miR-290 in mouse [21], which are known to play a key role in promoting maternal transcript turnover during maternal-zygotic transition. Furthermore, the expression pattern of miRNA-212 during early embryogenesis is inversely correlated with FIGLA expression during early embryogenesis, such that miR-212 expression increases steadily from 2-cell to 8-cell stage of embryogenesis, while FIGLA expression decreases gradually during the same period. The inverse correlation between miR-212 and FIGLA expression supports that miR-212 might be a post-transcriptional regulator of FIGLA during MET.

### Effect and specificity of miRNA-212 action on FIGLA expression

**Figure 5:**
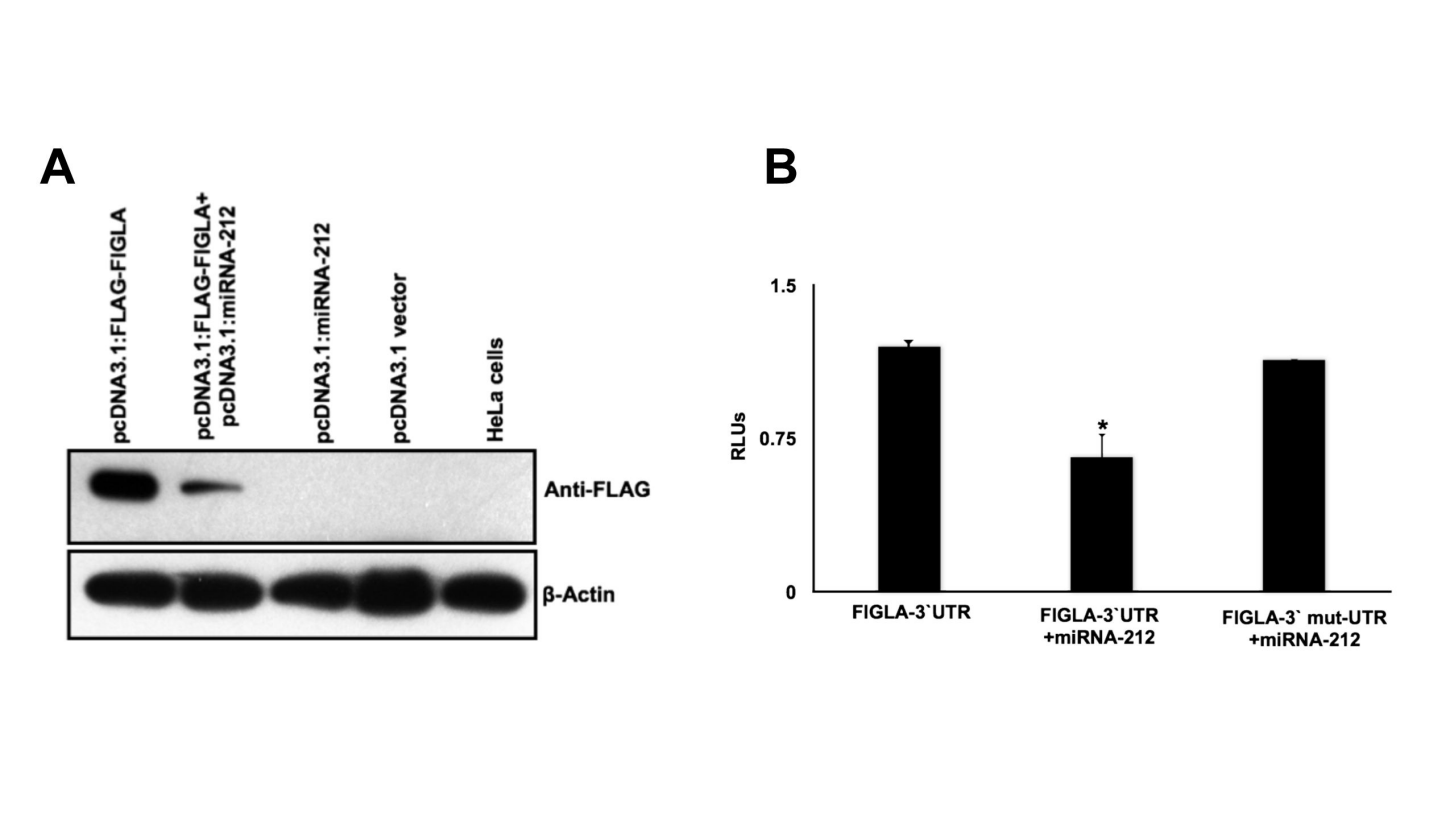
miR-212 specifically regulates bovine FIGLA *in vitro* in HeLa cells. (A) Western blot showing significant down-regulation of bovine FIGLA protein by ectopic expression of miR-212 in HeLa cells. β-Actin was used as a loading control. (B) Repression of luciferase activity due to specific interaction between miR-212 and the predicted MRE in the *FIGLA* 3`UTR cloned in the luciferase construct. Repression was abolished when the MRE was mutated. Relative firefly luciferase values were determined by the ratio of firefly to renilla luciferase with the negative control (cells transfected with native *FIGLA* 3`UTR construct alone) set at 1. Each group represents the mean ± SEM of four wells for an experiment repeated six times with similar results.

In order to test if miR-212 is indeed capable of regulating FIGLA protein expression, we used the human cervical cancer cell line HeLa because it expresses neither FIGLA nor miR-212 (data not shown). HeLa cells were grown in culture and transiently transfected with pcDNA3.1: FLAG-FIGLA and either pcDNA3.1: miR-212 or empty pcDNA3.1 vector as a negative control. Forty-eight hours after transfection, protein extracts were prepared. Western blot analysis with an antibody against FLAG shows a significant inhibition of FLAG-tagged FIGLA protein expression in cells expressing miR-212 compared to the control cells without miR-212 (Figure 5A), indicating that translation of FIGLA is repressed by miR-212. Furthermore, in order to validate the specificity and the efficacy of miR-212 action through the predicted binding site; we inserted *FIGLA* 3’ UTR sequence downstream of the firefly luciferase-coding region. A four-base pair mismatch mutation was introduced in the predicted MRE in the 3’ UTR of the *FIGLA* for miR-212 such that interaction between miR-212 and *FIGLA* mRNA was compromised. Ectopic expression of miR-212 suppressed the activity of the luciferase construct containing the miR-212 binding site of *FIGLA* mRNA at its 3’ end. Suppression of luciferase activity was abolished when the mutation was introduced into the seed region of the miRNA-212 recognition sequence in the *FIGLA* 3’ UTR (Figure 5B), indicating that the predicted MRE is critical for the direct and specific binding of miR-212 to *FIGLA* mRNA. Taken together, these data indicate that miR-212 binds to the 3’ UTR of *FIGLA* mRNA and impairs *FIGLA* mRNA translation.

### Effect of miR-212 on FIGLA expression in early embryos

Abundant evidence supports an important role for miRNAs for vertebrate embryogenesis. The dynamic temporal expression changes of miRNA in pre-implantation embryos and the increased synthesis of miRNA after the 2-cell stage in mouse embryos suggests that miRNA have a functional role during early embryonic development [21]. These findings were further supported by the observation that genetic ablation of Dicer, a miRNA-processing enzyme, in mouse and zebrafish embryos abolished the production of all mature miRNAs and endogenous siRNAs and resulted in embryonic lethality [18,20,44]. In contrast, targeted deletion of oocyte DGCR8, a RNA-binding protein required specifically for miRNA processing, had no significant effect on the maturation of oocytes and preimplantation development, even though miRNA levels were reduced to similar levels as Dicer-deficient oocytes [45]. This suggested that miRNAs are dispensable for oocyte maturation and early development. Recent studies have demonstrated that miRNAs could also be generated by direct transcription of short hairpin RNAs through noncanonical miRNA pathways, which are either DGCR8 independent-Dicer dependent or DGCR8 dependent-Dicer dependent [46]. It is thus still unknown if miRNAs generated via these pathways play a role in oocyte and embryo development. Further, inherent species-specific differences in the spatio-temporal expression and specific functional roles of miRNAs during early development cannot be discounted. Given that studies of posttranscriptional regulation during development have been limited to select model organisms, a more broad analysis of the function of miRNAs is required to better understand this aspect of evolution.

**Figure 6:**
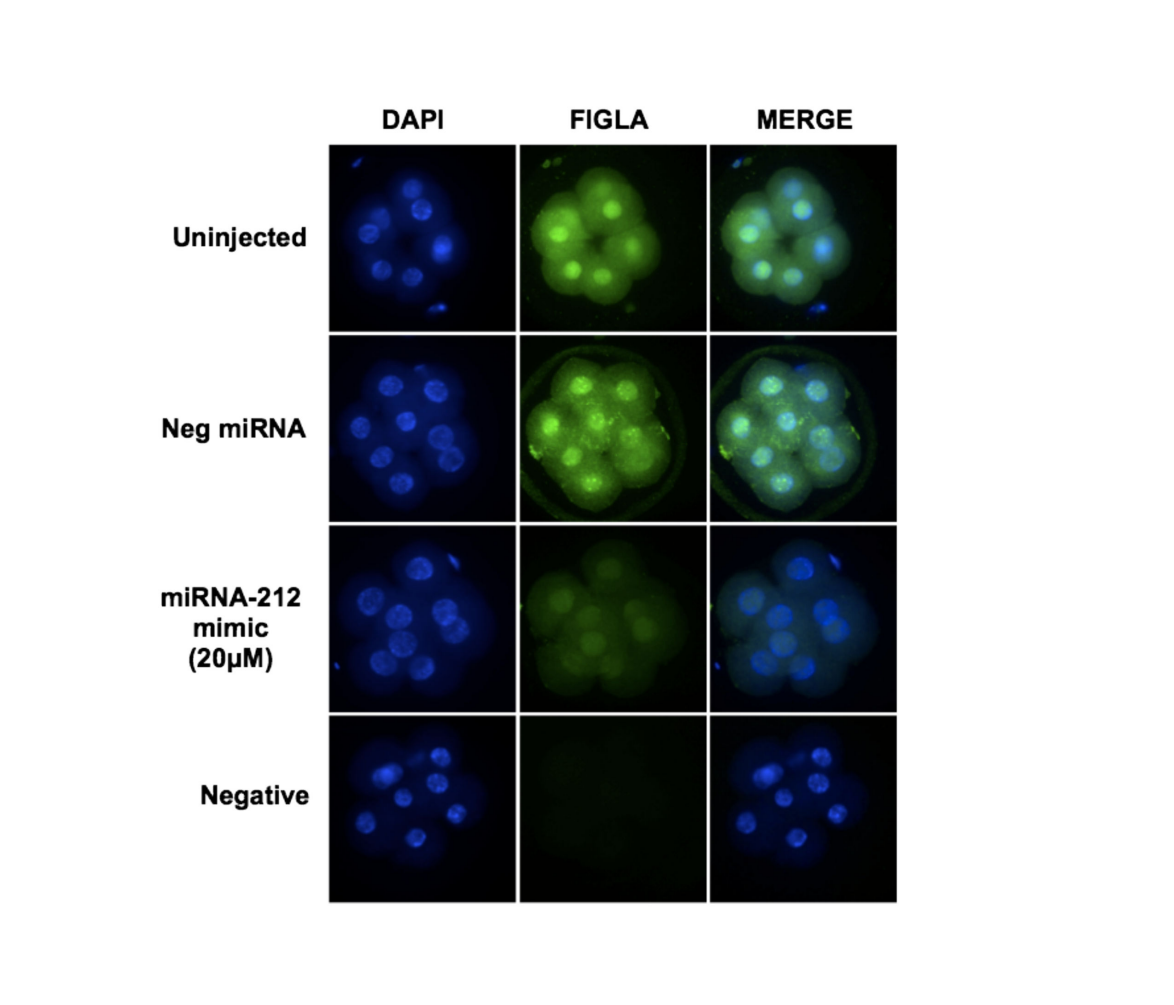
miR-212 represses endogenous FIGLA expression in early embryos. Effect of miR-212 mimic microinjection on abundance of FIGLA protein in 8-cell embryos as determined by immuofluorescent staining with anti-FIGLA antibody (n = 2 pools of 20 embryos per treatment). Uninjected embryos and embryos injected with a nonspecific miRNA (cel-miR-67N) mimic were used as controls. Nuclear DNA was stained with DAPI.

Studies in zebrafish, flies and Xenopus demonstrated that miRNAs are essential for the degradation of maternal transcripts during maternal to zygotic transition. In particular, zebrafish miR-430 and Xenopus miR-427 (an orthologue of miR-430) are highly expressed at the onset of zygotic transcription and facilitate the deadenylation and clearance of maternal mRNAs during early embryogenesis [18,19]. Despite these progresses, it is still relatively unknown how miRNAs regulate maternal transcripts in mammalian embryos. In particular, the *in vivo* targets of miRNAs are largely unknown. So far, very few studies have investigated the role of miRNAs during the bovine maternal-to-zygotic transition. Recently we demonstrated that miR-196a and miR-181a function as negative regulators for two key oocyte-specific maternal effect genes (*NOBOX and NPM2*) essential for early embryogenesis [33] [29]. Since we have shown that miR-212 is capable of regulating FIGLA expression through direct binding to the 3’ UTR of its mRNA and that miR-212 is differentially expressed during early embryogenesis, we next investigated whether miR-212 regulates FIGLA expression in early embryos. Microinjection of miRNA mimics into the zygotes has been the most commonly used approach in zebrafish, Xenopus and mice to determine the function of specific miRNAs and experimentally validate its targets [47–49]. As shown in Figure 6, microinjection of miR-212 mimic into bovine embryos effectively reduced FIGLA protein expression in 8-cell embryos compared to the uninjected and the negative control miRNA mimic injected embryos. Previous studies have demonstrated that the length of the poly (A) tail is the determining factor for the translational potential of maternal RNA transcripts. Elongation of poly (A) is associated with an increase in translation, whereas translational repression correlates with the shortening of the poly (A) tail [50]. Studies in zebrafish and Xenopus illustrated that miRNAs mediate the rapid deadenylation involving shortening of the poly (A) tail at the 3’ end of the maternal mRNAs and induce their degradation during early embryogenesis [18,19]. These results indicate that miRNA-induced clearance of maternal mRNAs might be an evolutionary conserved universal mechanism during early embryogenesis. Although such direct evidence from mammals is limited, we hypothesize a similar mechanism is likely to be involved in the negative regulation of FIGLA by miR-212 in bovine embryos during MET.

miR-212 and miR-132 are evolutionary conserved tandem miRNAs, well known for their essential role in the development, maturation and function of neurons [42]. Deregulation of miR-212/132 has also been associated with several neurological disorders, such as Alzheimer’s disease and tauopathies [51]. Besides their multiple roles in neuronal development, increasing evidence point towards an important involvement of miR-212 and miR-132 in mediating many other biological processes, including inflammation [52], immune function [53] and other cellular dysfunctions such as cancer. The expression of miR-212 has been reported in different kinds of tumors. In human lung cancer [54], pancreatic cancer [55], breast cancer [56] and colorectal cancer [57], miR-212 expression was significantly up regulated, whereas in osteosarcoma [58] and gastric cancers [59], the expression of miR-212 has been shown to be down-regulated. Furthermore, recent evidence has demonstrated that miR-212 and miR-132 play an important role as post-transcriptional regulators in granulosa cells [60]. Following LH/hCG stimulation in the ovarian cells miR-132 and miR-212 were found to be highly up regulated and computational analysis has identified nearly 77 putative mRNA as potential targets of miR-212 and miR-132 in granulosa cells [60]. The different levels of expression of these miRNAs in various developmental processes and disorders highlight their crucial role in regulating gene expression controlling diverse cellular and metabolic pathways. To our knowledge, no one has investigated the role of miR-212 during early embryogenesis. Our results support and unveil a novel potential role for miR-212 in regulating maternal mRNAs during early embryogenesis.

## Conclusion

Collectively, our results provide novel information regarding the temporal expression of FIGLA during bovine oocyte maturation and early embryonic development. We also provide several lines of evidence to support a new role for miR-212 as a bona fide negative regulator of FIGLA during early embryogenesis. First, the expression of miR-212 is inversely correlated to the expression of FIGLA during bovine early embryonic development. Second, miR-212 represses the expression of bovine FIGLA protein in HeLa cells. Third, miR-212 suppresses the activity of a luciferase reporter fused with the 3`UTR of FIGLA in a MRE dependent manner, and finally, ectopic expression of miR-212 mimic in bovine early embryos significantly represses FIGLA protein expression. Our report is the first to identify a miRNA that directly regulates FIGLA and display a novel potential role for miR-212 during early embryogenesis.

## Supporting Information

Figure S1 **Cloning of bovine FLGLA cDNA.** (A) Schematic illustration of two isoforms of bovine FIGLA mRNA. Isoform 2 results from alternative splicing of exon 4. (B) Confirmation of two isoforms by RT-PCR analysis. PCR was performed using primers flanking exon 4 and PCR products were separated by 3% agarose gel electrophoresis.
(DOC)

Figure S2 **Multiple alignment of deduced amino acid sequences of bovine (bFIGLA-1 and bFILGA-2), human (hFIGLA) and mouse (mFIGLA) FIGLA proteins by ClustalW analysis.** The conserved helix-loop helix domain is boxed.
(DOCX)

Table S1 **List of primers used in the study.**
(XLSX)

Table S2 **Genomic organization of bovine *FIGLA* gene.**
(XLSX)
