# Supplementary figures and images for: MicroRNA-212 Post-Transcriptionally Regulates Oocyte-Specific Basic-Helix-Loop-Helix Transcription Factor, Factor in the Germline Alpha (FIGLA), during Bovine Early Embryogenesis
Source: PLoS One. 2013 Sep 27;8(9):e76114. doi: 10.1371/journal.pone.0076114 (PMC3785419; doi:10.1371/journal.pone.0076114)

**Figure S2.**


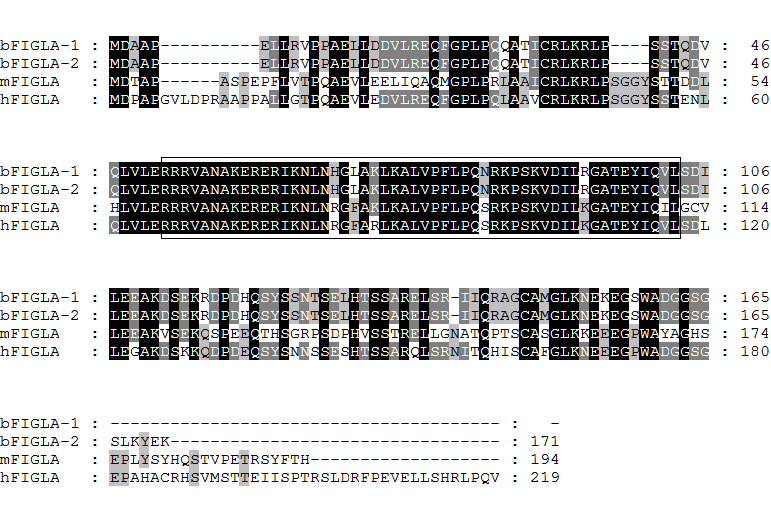

Supplement: Figure S2 — Multiple alignment of deduced amino acid sequences of bovine (bFIGLA-1 and bFILGA-2), human (hFIGLA) and mouse (mFIGLA) FIGLA proteins by ClustalW analysis. The conserved helix-loop helix domain is boxed. (DOCX) [file pone.0076114.s002.docx]
